# The impact of the national action plan on the epidemiology of antibiotic resistance among 352,238 isolates in a teaching hospital in China from 2015 to 2018

**DOI:** 10.1186/s13756-019-0473-y

**Published:** 2019-01-29

**Authors:** Shanjuan Wang, Yanhong Jessika Hu, Paul Little, Yifei Wang, Qing Chang, Xudong Zhou, Michael Moore, Joseph Irvin Harwell

**Affiliations:** 1 Shanghai General Practice Medical Education and Research Center, Jiading District Central Hospital Affiliated Shanghai University of Medicine & Health Sciences, No 1 Chengbei Rd, Jiading, Shanghai, 201800 China; 2 School of Public Health, The University of Hong Kong, G/F, Patrick Manson Building (North Wing), 7 Sassoon Road, Pokfulam, Hong Kong; 3 Primary Care and Population Sciences, University of Southampton, Aldermoor Health Centre, Aldermoor Close, Southampton, SO16 5ST UK; 4 School of Public Health, Zhejiang University, 866 Yuhangtang Road, Hangzhou, 310058 Zhejiang China; 5 Clinical Science Team, Clinton Health Access Initiative, 383 Dorchester Ave, Boston, MA 02127 USA

**Keywords:** National action plan, Epidemiology, Antibiotic Resistance Hospital, China

## Abstract

**Background:**

We sought to understand the epidemiology and characteristics of antimicrobial resistance (AMR) and the impact of the National Action Plan (NAP) on AMR. This information will be critical to develop interventions and strengthen antibiotic stewardship in hospital settings in China.

**Methods:**

Cross-sectional data collection from the hospital information management system from 1 January 2015 to 30 August 2018. Variables included patient age, sex, diagnosis, hospital department and antibiotic sensitivity test. T-test for two samples method was applied to compare the results before and after NAP implementation. Multivariate analysis with binary logistic regression was conducted to examine the associations of risk factors for antimicrobial resistance.

**Results:**

In total there were 352,238 isolates in the final analysis after excluding contamination strains and isolates with incomplete information. More than 50% of patients were > 66 years old. 62% were male. 40% of the total samples were sputum. Among the total sample, the total resistance rate was 42% among all isolates. The rate of resistance to all antibiotics declined by 5.3% (95% CI 4.96–5.64%, *p* < 0.0001) and culture positivity rate declined by 9.8% (95% CI 9.22–10.34%, *p* < 0.0001) after NAP. Logistical regression showed that the NAP had effect with an adjusted odds ratio of 0.76 (95% CI 0.71–0.81, *p* = 0.002). Being male, age > 65 years, ICU department, diagnosed with certain diseases were more likely to be associated with antimicrobial resistance.

**Conclusions:**

Antibiotic resistance rates were high in this teaching hospital. However, the introduction of the China NAP since 2016 followed by hospital policy emphasis was associated with a declining AMR trend. Policies will need to incorporate antimicrobial stewardship with a focus on certain departments, with infection control practices and with increases in vaccination coverage among elderly.

## Background

Antimicrobial resistance (AMR) has been recognized as a major public health crisis for decades as it is associated with adverse effects on morbidity, mortality, and economic costs including longer and costlier hospital stays [1, 2].

Between 2005 and 2014 AMR rates in China increased with the emergence of some complicated resistance patterns, such as extended-spectrum b-lactamase (ESBL)-producing isolates resistant to 3rd generation of cephalosporins increasing from 52.2 to 63.2%; *Klebsiella* with resistance to carbapenems from less than 3% to more than 10%; *Acinetobacter baumannii* resistant to cefoperazone/sulbactam and minocycline increasing from 25 to 37.7% and from 33 to 49.7%, respectively [3]. In 2015, the World Health Organization (WHO) launched the global national action plan (NAP) to combat antimicrobial resistance. In 2016, China also launched their NAP to Contain Antimicrobial Resistance (2016–2020), the key interventions of which were related to hospital settings including: 1) To standardize management of use of antibacterial agents and implement antimicrobial stewardship programs; 2) To optimize antimicrobial consumption and antimicrobial resistance surveillance network in clinics; 3) To enhance the training of professional personnel in rational use of antimicrobials and antimicrobial resistance; 4) To establish antimicrobial resistance reference laboratories and bacterial strain banks [4, 5]. The G20 declared its support for the fight against antimicrobial resistance in 2017 in Hangzhou China [6]. The Chinese national AMR surveillance system reported prevalence data [5]. However, resistance rates among some of the critical Gram negative bacteria are still rising despite the current efforts [7]. Whether the NAP has had an impact on individual hospitals is unclear. In this study we investigated the prevalence of bacterial isolates and antimicrobial resistance to understand how to better target programs for AMR control in Chinese hospital settings.

## Methods

### Patients and samples

Microbiology and antibiotic susceptibility was recorded from all isolates collected at the hospital from 1 January 2015 to 30 August 2018. All data were retrospectively reviewed using the hospital medical record system. Data extracted from the system for each isolate included demographic characteristics of the patient (age, sex), diagnoses (such as hypertension, diabetes, pneumonia and other diseases), sample type and department of the hospital obtaining the sample. Samples from blood, sputum, urine, stool, and others including tissue, fluid or deep wound cultures obtained during operations, abdominal drains, fluid from paracentesis or percutaneous aspiration of abscesses, and those from drain bottles or superficial wounds were included. Those from air or operational surfaces, health care provider hands and white coats were excluded. The study protocol was approved by the Institutional Review Board Ethics Committee of Jiading Hospital.

### Microbiological examination and antibiotic susceptibility determination

Data was collected from the microbiology laboratory from 2015 to 2018. The identification of bacterial species was performed according to the criteria of the American Society for Microbiology [8], all bacteria isolated were included in the study. Species identification of the isolates was performed by standard biochemical methods, the API 20E system or the Vitek 2 automated system (bioMérieux, Marcy l’Etoile, France). Antimicrobial susceptibility tests and the minimum inhibitory concentrations (MIC) of antibacterial agents were evaluated by the agar dilution method (bioMérieux, France) according to Clinical and Laboratory Standards Institute (CLSI, Wayne, PA) guidelines [9]. Antibacterial susceptibility testing for the most commonly used antibiotics for a given microorganism was routinely performed for all potential pathogens isolated from any sample site. Samples from the same patient within 3 days except for contaminated samples was not repeated when a microorganism was isolated more than once. All isolates for antibacterial susceptibility testing had been performed were recorded in a computer database. All frequent Gram-positive and Gram-negative bacteria and for the most commonly prescribed antibiotics in the hospital were included, such as penicillin (piperacillin, amoxicillin, ampicillin, oxacillin amoxicillin/clavulanate, piperacillin/tazobactam, ticarcillin/clavulanate); cephalosporin (ceftriaxone, ceftazidime, cefepime, cefuroxime, cefotaxime, cefoxitin, cefazolin, ceftazidime/clavulanic acid); aminoglycoside (amikacin, gentamicin, tobramycin); fluoroquinolone (levofloxacin, ciprofloxacin, oxacillin, moxifloxacin,); macrolide (josamycin, azithromycin, roxithromycin, erythromycin); carbapenem (ertapenem, meropenem, imipenem); lincomycin (lincomycin, clindamycin), sulfonamide (trimethoprim/sulphonamide); tetracycline (doxycycline, tetracycline, minocycline), peptidoglycan (fosfomycin), colistin, chloramphenicol, oxazolidinone, monobactam, vancomycin, nitrofurantoin. The MICs at which 100% of the isolates were inhibited (MIC100) with external inter-laboratory quality control were determined. The detail of the MIC reference ranges is provided in the supplemental materials. The rate of antimicrobial resistance refers to the number of resistant isolates divided by the total number of isolates. The level of resistance included high and intermediate level resistance, low level resistance was not included.

### Statistical analysis

Results pertaining to patients’ clinical characteristics and antimicrobial resistance are expressed as a percentage of samples. Differences between the periods before (January 2015–August 2016) and after NAP (September 2016–August 2018) were statistically analysed using the *× 2/t* test with Stata 14 (StataCorp LLC). Multivariate analysis with binary logistic regression was conducted to examine the associations of risk factors with control for potential confounders, adjusted odds ratio [8] was applied in this study [8, 10]. All variables with a *P*-value of < 0.05 in the univariate analysis were included in the logistic regression model. A two-tailed *P*-value of < 0.05 was considered to indicate statistical significance.

## Results

### Characteristics of the isolates

In total there were 352,247 isolates from 2015 to 2018 in this study. 9 isolates had missing information which were excluded from the final analysis. 62% of the total isolates were from male patients, the mean patient age was 62 years old (SD ±0.036), more than half were over 66 years old. Most of the samples were from sputum and urine. The highest resistance rates were from sputum specimens, patients older than 81 years, male and located in the intensive care unit. Among total samples, the resistance rate was 42% with 74% high level of resistance isolates. Other characteristics are shown in Table 1.

**Table 1:** Characteristics of the total isolates and of them the antimicrobial resistance isolates from 2015 to 2018 (*n* = 352,238)

Items	No of samples	% among the category (column)	No with antimicrobial resistance	among the total (row)
Age				
0–21	9448	2.69	2643	0.28
22–49	89,382	25.48	30,176	0.34
50–65	72,377	20.63	33,503	0.46
66–80	95,712	27.29	45,487	0.48
81–102	83,840	23.9	42,277	0.50
Sex				
Male	216,792	61.84	104,175	0.48
Female	133,738	38.15	49,802	0.37
Year				
2015	91,056	25.85	41,498	0.46
2016	108,375	30.77	50,927	0.47
2017	103,038	29.25	43,579	0.42
2018	49,778	14.13	18,456	0.37
Season				
Spring	84,506	23.99	39,154	0.25
Summer	92,126	26.15	40,632	0.26
Autumn	100,197	28.45	41,045	0.27
Winter	75,418	21.41	33,638	0.22
Sample				
Blood	24,221	6.91	8421	0.35
Urine	63,673	18.15	27,742	0.44
Sputum	146,142	41.67	85,501	0.59
Catheter	3794	1.08	2156	0.57
Drainage	24,367	6.95	8053	0.33
Secretion	67,785	19.33	16,468	0.24
Body fluid	20,742	5.91	5685	0.27
Department				
ICU	72,224	20.57	49,919	0.69
Internal-dept.	189,495	53.98	77,298	0.41
Surgical-dept.	75,872	21.61	22,821	0.30
Outpatient -dept.	8936	2.55	1998	0.22
Emergency-dept.	4532	1.29	2106	0.46

Note: *dept.* department; body fluid includes pleural effusion, ascites, cerebrospinal fluid and uterine effusion

### Trends of culture positivity and antimicrobial resistance 2015–2018

**Fig. 1:**
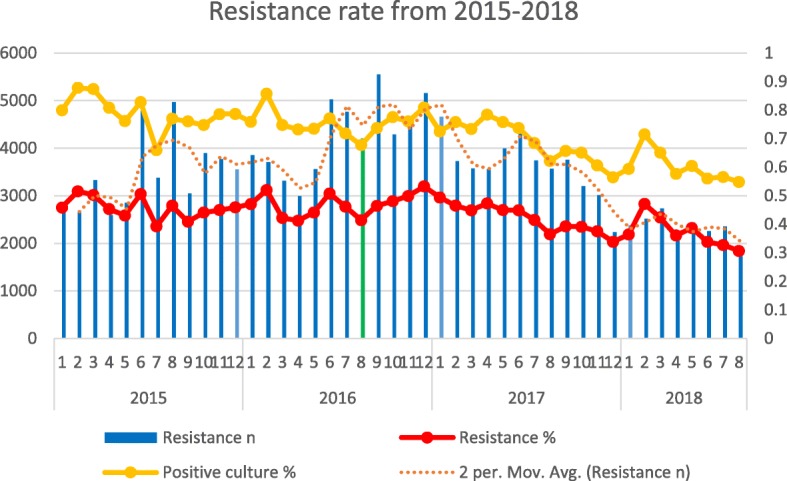
The trends of culture positivity and antimicrobial resistance rates from 2015 to 2018. Notes: Resistance *n* = absolute number of the resistance isolates; Resistance % = Resistance n/total number of the isolates at certain period; Positive culture *%* = absolute number of the positive culture/total number of cultures at certain period. The green line August 2016 is the time point of national action plan

There were 187,492 and 164,746 isolates obtained before and after NAP respectively. Overall the trends of culture positivity rates and antimicrobial resistance rates are similar. Both had declining trends with highest peaks in winter and summer as shown in Fig. 1.

### Changes in culture positivity before and after NAP

In bacteria cultures after NAP launched, total culture positivity rate changed from 41.3 to 31.5%, a reduction of 9.8% (95% CI 9.22–10.34%, *p* < 0.0001). The total Gram positive percentage was reduced from 80.6 to 72.5% after NAP while Gram negative percentage had a slight increase from 37.6 to 38.9%.

**Fig. 2:**
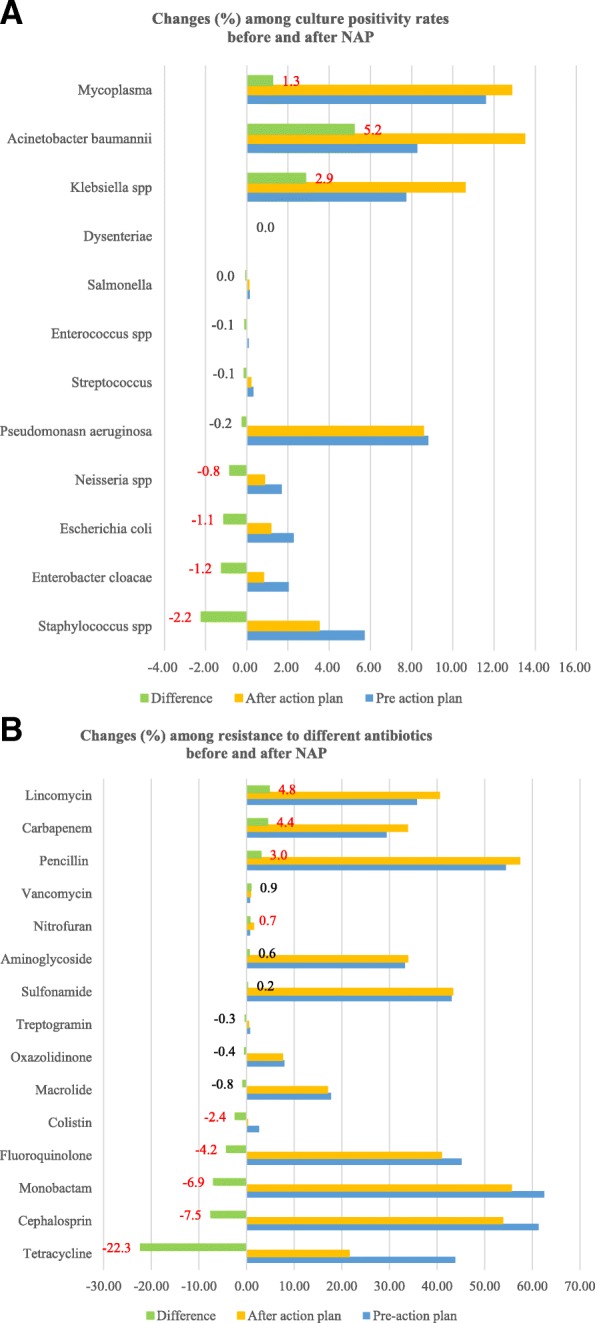
**a** Culture positivity percentage changes for different bacteria from 2015 to 2018 before and after action plan (NAP). Notes: the percentage = absolute positive cultures/total number of the cultures; Streptococcus spp. included Streptococcus A 21/451 = 0.5%, Streptococcus B 98/451 = 22%; Staphylococcus spp. included staphylococcus pneumonia 1076/2882 = 37%. The difference number in red with *p* value< 0.05. **b** The changes in antimicrobial resistance to different class of antibiotics from 2015 to 2018 before and after national action plan (NAP). Notes: Resistance rate (%) = Resistance n/total number of the isolates at relevant period; The difference number in red with *p* value< 0.05. Each category included one or several individual antibiotics: penicillin (piperacillin, amoxicillin, ampicillin amoxicillin/clavulanate, piperacillin/tazobactam); cephalosporin (ceftriaxone, ceftazidime, cefepime, cefuroxime, cefotaxime, cefoxitin, cefazolin, ceftazidime/clavulanic acid); aminoglycoside (amikacin, gentamicin, tobramycin ticarcillin/clavulanate); fluoroquinolone (levofloxacin, ciprofloxacin, oxacillin, moxifloxacin,); macrolide (josamycin, azithromycin, roxithromycin, eryphilin);carbapenem (Ertapenem, Meropenem, Imipenem); lincomycin (lincomycin,, clindamycin), sulfonamide (trimethoprim/sulphonamide); tetracycline (doxycycline, tetracycline, minocycline),colistin, oxazolidinone, monobactam, vancomycin, nitrofuran

The top three bacteria increases after NAP launched were *Acinetobacter baumanni* from 8.28 to 13.49%, a 5.2% increase; *Klebsiella pneumoniae* increased by 2.9% from 7.74 to 10.6%; *Mycoplasma* increased by 1.3% from 11.61 to 12.86%. The bacteria which declined were *Staphylococcus* spp. by 2.2%, *Enterobacter cloacae by 1.2%, Escherichia coli by 1.1%* and *Neisseria spp by 0.8%* as shown in Fig. 2a*.*

### Changes in antimicrobial resistance before and after NAP

The total resistance rate was 42% among all the isolates. The rate of resistance to all antibiotics declined by 5.3% (95% CI 4.96–5.64%, *p* < 0.0001), from 46.3 to 41% after NAP launched. Resistance to different antibiotic classes had varied changes. The rates of resistance to antibiotic classes declined as shown in the Fig. 2b for tetracycline by 22%, cephalosporins by 7.5%, monobactam by 6.9%, and fluoroquinolone by 4.2%. Resistance increased to nitrofurantoin by 0.7%, penicillin by up to 3%, carbapenem by 4.4% and lincomycin by 4.8%.

### Antimicrobial resistance rate changes among different diseases and departments

**Fig. 3:**
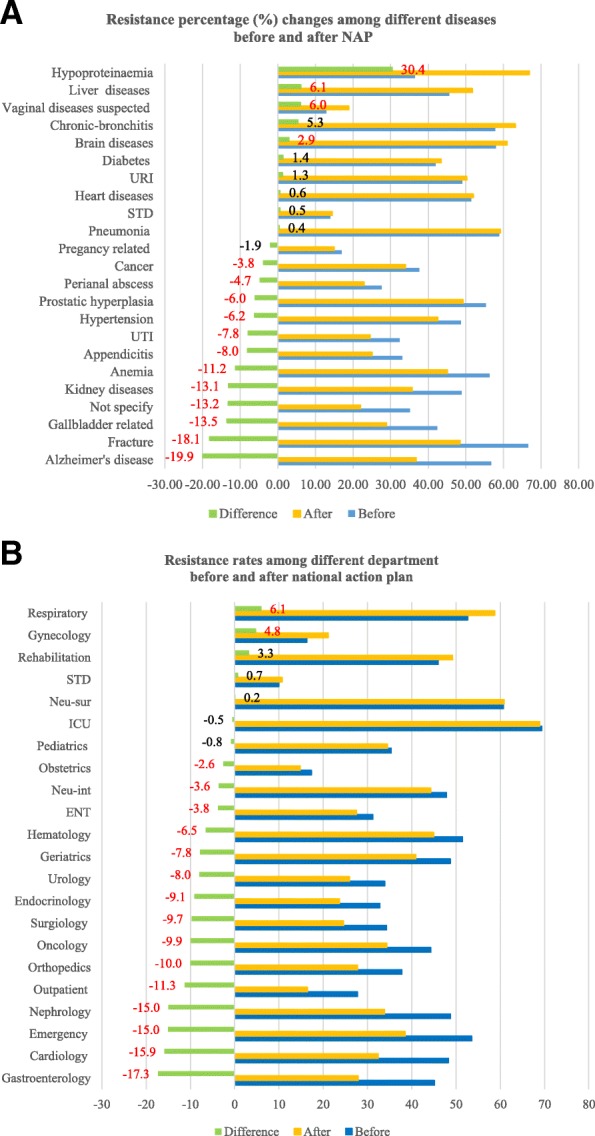
**a** Antimicrobial resistance percentage changes among different diseases before and after national action plan (NAP). Notes: *UTI* urinary tract infection, *URI* upper respiratory infection, *STD* sexually transmitted diseases; hypertension includes all types of hypertension (grade I, II, III, IV). The percentage number in red with *p* value < 0.005. **b** Antimicrobial resistance rates among different department before and after action plan. Notes: *ICU* intensive care unit, *Neu-sur* Neurosurgery, *Neu-int* Neurological internal medicine, *STD* sexually transmitted diseases. Other = non-direct-clinical department includes nutrition, infection control, laboratory, education & prevention departments. The percentage number in red was with *p* value < 0.05

We investigated the overall antimicrobial resistance rates among different diagnoses. The top 5 highest rates were among patients with hypertension, pneumonia, urinary tract infection, diabetes, and acute appendicitis; The increased rates among diseases with significant *p* value were hypoproteinaemia, liver diseases, vaginal diseases, brain diseases (included stroke, cerebral haemorrhage, brain injuries); other diseases such as pneumonia, sexually transmitted diseases (STD), heart diseases, upper respiratory infection (URI). Diabetes had slight increases but with insignificant p value. More than 10% reduction was seen with Alzheimer’s disease, fracture, gallbladder related diseases, kidney diseases and anaemia. Cancer, perianal abscess, prostatic hyperplasia, hypertension, urinary tract infections and appendicitis had reduction from more than 3% up to 8% as shown in Fig. 3a.

The intensive care unit submitted 20% of the total isolates, and of these up to 70% of the isolates were resistant. This was followed by the neurosurgery, respiratory, haematology, rehabilitation and emergency departments. The resistance rates for most departments declined after NAP. Gastroenterology, cardiology and emergency departments had reductions of 17% (95% CI 15.06–19.49%, *p* < 0.0001), 16% (95% CI 14.54–17.19%, *p* < 0.0001) and 15% (95% CI 12.16–17.91%, *p* < 0.0001) respectively. The gynaecology department experienced a statistically significant increase of 4.8% (95% CI 3.71–5.90%, *p* < 0.0001) and resistance in the respiratory department increased by 6.1% (95% CI 4.69–7.44%, *p* < 0.0001). The intensive care unit (ICU) and paediatric departments both had insignificant reductions while neurosurgery and rehabilitation departments had insignificant increases as shown in Fig. 3b.

### Risk factors for antimicrobial resistance

In the multivariate logistical regression, the adjusted odd ratios and *p*-values show that being female, age less than 49 years, not in ICU department, diagnosed with diabetes, perianal abscess, urinary tract infection were less likely to be associated with antimicrobial resistance as shown in Table 2. The NAP had an associated effect with aOR 0.76 (95% CI 0.71, 0.81, *p* = 0.002) as seen in Table 2.

**Table 2:** Multivariate logistical regression on antimicrobial resistance before and after action plan (*n* = 352,238)

Items	aOR	95% CI	*p*
Sex			
Female	0.87	0.85–0.89	< 0.001
Male	1		
Age			
0–49	1		
50–80	1.12	1.09–1.16	< 0.001
81–102	1.15	1.11–1.19	< 0.001
Department			
ICU	1		
Ward	0.38	0.37–0.39	< 0.001
Outpatient clinics	0.32	0.31–0.34	< 0.001
Sample type			
Blood	1		
Urine	1.51	1.44–1.57	< 0.001
Sputum	1.74	1.66–1.81	< 0.001
Other	0.8	0.77–0.84	< 0.001
Diseases			
Hypertension	1		
Hypoproteinemia	2.36	2.12–2.63	< 0.001
Pneumonia	1.39	1.34–1.45	< 0.001
Chronical-bronchitis	1.44	1.33–1.56	< 0.001
Anemia	1.25	1.15–1.36	< 0.001
Brain diseases	1.22	1.17–1.27	< 0.001
Fracture	1.18	1.09–1.27	< 0.001
Prostatic hyperplasia	1.18	1.12–1.25	< 0.001
Appendicitis	1.09	1.03–1.45	0.002
Heart diseases	1.04	0.88–1.46	0.135
Kidney diseases	0.98	0.92–1.04	0.509
Diabetes	0.94	0.90–0.99	0.013
Perianal abscess	0.92	0.84–1.0	0.043
Urinary tract infection	0.76	0.72–0.81	0.002
Group			
Before NAP	1		
After NAP	0.79	0.77–0.80	< 0.001

Notes: Other = catheter, drainage, secretion and body fluid. NAP = national action plan

## Discussion

This study, from a large sample of isolates during a 44 month period, shows a high level of resistance at 42% of the total isolates. The average culture positivity rate was 28%, which is similar to other studies in China [11, 12]. The total resistance declined by 5.3% and culture positivity rates declined by 9.8% after the introduction of the NAP. This is the first study to show the relationship between the NAP and an individual hospital AMR rates. The changes in resistance rates varied between departments with the greatest reductions in gastroenterology, cardiology, emergency and outpatients departments. Among different diseases, the greatest reductions were seen with Alzheimer’s disease, fracture and gallbladder-related diseases. The patients with the highest rates of resistance were those with brain diseases, and most of the resistant strains were from sputum and urine. Being older than 80 years, male, in the ICU department and suffering from hypoproteinaemia, pneumonia, chronic bronchitis, anaemia, fracture, prostatic hyperplasia, or appendicitis were more likely associated with bacterial resistance.

Gram positive bacteria had resistance rates of over 35% in this study before and after NAP, which is higher than the national rate of 29% in 2016. At the same time, the Gram negative resistance rate was 88% before NAP, after NAP, the rate was still slightly higher than the national rate of 71% in 2016 [13]. Our study differs from the national surveillance data, which involves more than 1000 hospitals from 31 provinces, in that national surveillance is limited to the top 5 bacteria whereas we included all isolates in a single hospital [7]. A recent study from FP Hu et al. found total resistance rates from 52 to 60% between 2005 and 2014 [3], which is higher to the total resistance rate of 42% in this study. Our study had similar trends for increases in *Acinetobacter baumanni* and *Klebsiella pneumoniae,* while we found *Enterobacter cloacae* and *Staphylococcus* spp. had declines which similar to the national trends for these two bacteria [13].

The trends for the culture positivity and antimicrobial resistance both had seasonal trends, with peaks in winter and summer. This also in line with the antibiotic prescribing patterns reported in another nearby hospital [14], and corresponds with the influenza season in Shanghai [15]. Influenza vaccination has a very low coverage in the elderly in Shanghai according to a study of the vaccination rate in 2016–2017 [16]. In our study the highest resistance rates were concentrated among those older than 80 years. This might contribute to the seasonal trends among elderly. Several studies have shown the effectiveness of influenza vaccination in reducing antimicrobial use and antimicrobial resistance [17, 18]. Similar evidence exists for pneumococcal conjugate vaccine (PCV) use in older adults, which can reduce episodes of pneumonia [19]. Further study on vaccination to reduce AMR in this city is needed.

We found an increase in resistance to antibiotics among patients diagnosed with hypoproteinaemia, liver diseases, anaemia, fracture, prostatic hyperplasia, or appendicitis. Many patients with hypoproteinaemia were from the ICU, where a high prevalence of resistance to multiple antibiotics was found. Nevertheless one study from China showed hypoproteinaemia and anaemia were independent risk factors for acquiring AMR in ICU department [20]. Another study found patients with liver diseases had a high prevalence of AMR in China [20], which is similar to our study. A study of acute appendicitis that reported high rates of AMR that were community-acquired infections [21]. Further study on the association of higher AMR among specific diseases is needed to explore the prevention component of antimicrobial stewardship in this hospital. As the diagnoses were extracted from the electronic health record system, future study on the validation and accuracy on diagnosis might be necessary.

The resistance rates declined over time among all the isolates for tetracycline, monobactam and cephalosporin, while increasing for carbapenem, penicillin and aminoglycoside. The observed declines may have been the result of the recent national policy on reinforcing antimicrobial resistance control as part of the assessment for hospital merit [5, 22]. The big drop in tetracycline resistance may be related to a general decrease in this drug’s use in this hospital for several years. Whether a decline in tetracycline resistance is also related to a reduction in tetracycline use in food animal production and environmental contamination after the NAP remains unknown but it is noteworthy that a recent study found high concentrations of tetracycline residuals among children’s urine in the same city [23, 24]. Further studies on animal antimicrobial use in China and AMR are urgently needed.

Increases in carbapenem resistance represents an important threat to clinical care as carbapenems are an important group of last-line antibiotics for treating multidrug-resistant Gram-negative bacteria such as *K. pneumoniae* and *E. coli* [25]. This increase might be the result of increased access to medicines and increased opportunities for acquisition of resistance from other patients. This trend was also seen in Greece [26]. With high levels of multi-drug resistance, including resistance to carbapenems, few therapeutic options are available, and yet there are already examples of colistin resistance in China [27]. A study by Qu XY documented the increasing consumption of carbapenems in China [28]. Resistance to cephalosporin, monobactam and fluoroquinolone has reduced in this study which may be the result of earlier emphasis on appropriate use of these antibiotics and the higher resistance rates to those antibiotics in the past years [29]. Antibiotic resistance rates are related to antibiotic prescribing and use. In this study we did not look at the patterns of antibiotic use in the whole hospital. This will be the subject of future study. A study by Wang Y in another hospital in Shanghai showed the highest prescribing rates of antibiotics were for cephalosporin (54%) followed by macrolides/lincosamides and fluoroquinolones [14].

High rates of resistance in intensive care is not surprising as ICU usually has the most critically ill patients with an urgent need for antibiotics [30]. Several studies have shown high rates of resistance in ICUs, and multi-drug resistant organisms like *Acinetobacter, Klebsiella* and *Pseudomonas aeruginosa* are commonly seen in ICU patients [31, 32]. These bacteria had an increased trend in this study. A meta-analysis found that a history of ICU stay was associated with acquisition of resistant bacteria [33]. Though the total resistance rate had a slight reduction in ICU, absolute resistance rates still remain very high at up to 60% even after the NAP.

Resistance in the emergency and outpatient departments reflects the resistance rates in the community. The reductions of 15% in emergency and 11% in outpatients suggests that the NAP policy has not only impacted the hospital system but also the community level of resistance rates. Studies showed that the emergency and ICU department had high antibiotic prescription rates [14, 34]. This indicates that these departments should be the target for the future antimicrobial stewardship and infection control programs, and including hand hygiene and chlorhexidine body-washing, which has been shown to reduce acquired antimicrobial resistance in the ICU [35]. Available rapid laboratory tests for diagnosis and antibiotics selection would also increase appropriate antibiotic use and reduce antimicrobial resistance in emergency department [36, 37]. The reduction of AMR rate in emergency and outpatient departments might be due to increased awareness of this public health issue in recent years. This would suggest intervening to control the spread of antimicrobial resistance in community is important. The highest observed reduction of resistance rates was among gastroenterology, cardiology and nephrology departments, which might be the result of reinforcement of the standardized invasive clinical procedures, as well as appropriate antibiotic use.

This study showed a reduction both in culture positivity and resistance to antibiotics although the degree of the reduction was less than 10% after the launch of NAP. The impact of this reduction may be expected to reduce the economic burden. A recent study from Europe documented the scale of deaths and DALYs attributable to AMR [38]. Based on this evidence, the modest reduction from this study may have saved more than 400 deaths during this period.

Hospital policy was to provide continuous training on antimicrobial resistance for doctors beginning in 2015 although there was not a specific antimicrobial stewardship program in place for monitoring antibiotic use and resistance rates. Hospital documents and discussion with the hospital doctors indicate that a monitoring and scoring system for device infection control and surgical procedure was implemented since 2016 [39]. This might also have contributed to the observed reductions in resistance, especially in departments engaged in a large number of invasive procedures.

This study is not without limitations. First, the use of isolates rather than cases would tend exaggerate real resistance rates, since individuals, especially those with extensive resistance may have multiple samples collected. The population level of resistance remains unknown. Second, the interpretation of the antibiotic susceptibility results may vary from one laboratory technician to another. This laboratory has a CLSI standardized reading method, which should minimize this variability. Third, the diagnoses reported may not be accurate. However, the use of an electronic medical record should minimize entry errors and is the most accurate strategy for a retrospective study. Fourth, as we lacked antibiotic prescribing information, we cannot link resistance rates to prescribing rates. Even so, much antibiotic prescribing likely happens before entering the hospital as the high resistance rates from emergency department illustrate, and we would not be able to capture any of this pre-hospital data. Future study will focus on this variable in a prospective manner. Fifth, it is difficult to separate other policy contributions as there have been other policies before NAP [5, 22]. Lastly, As the study was done in a teaching hospital, this study cannot reveal and compare the precise extent of AMR before and after the NAP at community level. Further investigation including molecular-level diagnostics with collection of broad risk factors from different parts of the country is needed to generalize the national level of impact.

### Conclusion and recommendation

In conclusion, we have described the culture positivity and antimicrobial resistance rates in a public hospital in Shanghai after the introduction of the NAP to Contain Antimicrobial Resistance (2016–2020). Although the rates still remain high and some of the resistance is still increasing, overall the trends are declining. Future interventions should focus on the elderly, neurosurgery, ICU, respiratory and gynecology departments as well as the community, concentrating on the use of handwashing, chlorhexidine, rapid laboratory testing, and increasing vaccination rates. Further antimicrobial stewardship with appropriate components should be in place for further controlling AMR.

## Abbreviations

AMR: Antimicrobial resistance
CLSI: Clinical and Laboratory Standards Institute
DALY: Disability-adjusted life year
ICU: Intensive care unit
MIC: Minimum inhibitory concentrations
NAP: National Action Plan
PCV: Pneumococcal conjugate vaccine
STD: Sexually transmitted diseases
URI: Upper respiratory infection
WHO: World Health Organization
